# Effects of leaf traits of tropical trees on the abundance and body mass of herbivorous arthropod communities

**DOI:** 10.1371/journal.pone.0288276

**Published:** 2023-11-07

**Authors:** Jana E. Schön, Yvonne Tiede, Marcel Becker, David A. Donoso, Jürgen Homeier, Oliver Limberger, Jörg Bendix, Nina Farwig, Roland Brandl

**Affiliations:** 1 Department of Biology, Animal Ecology, Philipps-Universität Marburg, Marburg, Hesse, Germany; 2 Department of Biology, Conservation Ecology, Philipps-Universität Marburg, Marburg, Hesse, Germany; 3 Departamento de Biología, Escuela Politécnica Nacional, Quito, Pichincha, Ecuador; 4 Faculty of Resource Management, HAWK University of Applied Sciences and Arts, Göttingen, Lower Saxony, Germany; 5 Department of Geography, Laboratory for Climatology and Remote Sensing, Philipps-Universität Marburg, Marburg, Hesse, Germany; Baylor University, UNITED STATES

## Abstract

In tropical forests, herbivorous arthropods remove between 7% up to 48% of leaf area, which has forced plants to evolve defense strategies. These strategies influence the palatability of leaves. Palatability, which reflects a syndrome of leaf traits, in turn influences both the abundance and the mean body mass not only of particular arthropod taxa but also of the total communities. In this study, we tested two hypotheses: (H1) The abundance of two important chewer guilds (‘leaf chewers’ and ‘rostrum chewers’), dominant components of arthropod communities, is positively related to the palatability of host trees. (H2) Lower palatability leads to an increased mean body mass of chewers (Jarman-Bell principle). Arthropods were collected by fogging the canopies of 90 tropical trees representing 31 species in three plots at 1000 m and three at 2000 m a.s.l. Palatability was assessed by measuring several ‘leaf traits’ of each host tree and by conducting a feeding trial with the generalist herbivore *Gryllus assimilis* (Orthoptera, Gryllidae). Leaf traits provided partial support for H1, as abundance of leaf chewers but not of rostrum chewers was positively affected by the experimentally estimated palatability. There was no support for H2 as neither leaf traits nor experimentally estimated palatability affected the mean body mass of leaf chewers. The mean body mass of rostrum chewers was positively related to palatability. Thus, leaf traits and experimentally estimated palatability influenced the abundance and mean body mass of chewing arthropods on the community level. However, the data were not consistent with the Jarman-Bell principle. Overall, our results suggest that the palatability of leaves is not among the dominant factors influencing abundance and mean body mass of the community of chewing arthropod herbivores. If other factors, such as the microclimate, predation or further (a-)biotic interactions are more important has to be analyzed in refined studies.

## Introduction

Arthropod communities in tropical rainforests are highly diverse [1,2], interact in numerous, complex ways with their surrounding environment, and contribute to many ecosystem processes [3–6]. Herbivorous arthropods act as important drivers influencing plant growth, fitness, and survival [7–10] and thus shape both plant community composition (ecological time scale) and plant defense strategies (evolutionary time scale) that increase functional and phylogenetic diversity of plants [11–15]. Conversely, defense traits of plants influence the composition of arthropod communities [e.g., 16–18]. Thus, depending on the considered time scale, leaf traits can modify the fitness, abundance, and body size of arthropod individuals. This can be done by influencing their growth-, reproduction- and mortality rates and thus the abundance and composition of arthropod communities, e.g., with respect to feeding guilds [19–23]. Feeding guilds are characterized either by their mouthparts and the resulting damage to host plants or the plant parts that serve as food (e.g., leaf chewers or sap suckers) [24,25]. Among herbivorous feeding guilds, chewing arthropods, particularly Coleoptera, Orthoptera, and caterpillars of Lepidoptera and Hymenoptera, account for most of the foliar feeding damage in tropical forests [26–28], where the loss of leaf area due to arthropods ranges between 7% up to 48% [3,29].

Several leaf traits act on herbivores by influencing food quality, subsequently here termed palatability. Palatability can be either estimated by measuring the plant material consumed by an herbivore in feeding trials, by estimating the amount of leaf area loss caused by herbivores in natural systems, or by analyzing the influences of single or a number of specific leaf traits on herbivores. Here we concentrate on the first and last possibility. Palatability varies between food plant species and differs depending on the type of plant material consumed [16,30–33]. The food quality of leaves can be influenced by morphological leaf traits, (e.g., specific leaf area (SLA)); foliar nutrient concentrations (e.g., nitrogen (N), phosphorus (P), calcium (Ca), or potassium (K)); secondary plant metabolites (e.g., lignins); and the foliar accumulation of (heavy) metals (e.g., iron (Fe) or cadmium (Cd)) [e.g., 34–40] (S1 Table) also defined as elemental defense [41].

Although, SLA shows contradicting influences on palatability in literature [38,42,43] (S1 Table), it is often assumed to have positive effects since leaves with a great SLA have thin, large laminas and contain smaller amounts of poorly eatable structural compounds compared to small thick leaves [44]. Among the foliar nutrients, the N concentration tends to be positively related to palatability and arthropods populations [33,39,42,45] (S1 Table). The foliar nutrient P influences palatability depending on its concentration, leading to a relation following a hump-shaped curve [19,20,37] (S1 Table). Ca^2+^ ions play an important role to initiate herbivore induced defense mechanisms [46], however, it seems to have also positive influences on herbivorous arthropods [22,47] (S1 Table). The foliar nutrient K in contrast was predominantly identified to have negative influences on growth rates and survival of herbivorous arthropods in previous studies [40,48,49] (S1 Table). Lignins, as secondary plant metabolites reduce the palatability of leaves [50,51] since they form a mechanical barrier for herbivores [52,53] (S1 Table). Lastly, the heavy metals Cd and Fe are toxic for herbivores if accumulated in plants in high quantities [35,54,55], thus reducing the palatability, although the latter can have positive effects on herbivore development and reproductive fitness [22].

Jarman and Bell hypothesized that herbivorous ungulates of greater body mass can feed on food with a lower quality than species with a smaller body mass [30,56,57]. The Jarman-Bell principle is based on the observation that an animal’s energy (intake) per unit body mass increases with increasing body mass, with a slope of 0.75. Thus, per unit body mass, larger herbivorous mammals need less energy than smaller ones. This applies not only to vertebrates, but also to invertebrates like insects [58]. However, to compensate for low diet quality, food intake increases with animal body mass since the digestibility of consumed plants is not related to animal body mass [57,59–61]. This positive relationship between body mass and consumption rate was also recorded for several insect orders [62]. Nonetheless, this relationship does not give any indication about changes in the diet quality with changing consumption rates. However, several vertebrate groups follow the Jarman-Bell principle (e.g., ungulates [57,63–66], primates [67], carnivores [68], and reptiles [69]). Among arthropods, the Jarman-Bell principle seems to explain the diet of detritivorous termite species, whose body size increases with the relative amount of humus in the diet [70].

Body size is an important trait, with consequences for the energetics and physiology of individuals, the evolution of species, community structure, and ecosystem functions. The Jarman-Bell principle predicts that, on average, larger species consume low-quality resources. Apart from early studies on ungulate communities [57,64,65] all analyses of the Jarman-Bell principle were done at the species level. An extension of the Jarman-Bell principle to arthropod communities leads to the prediction that phytophagous insect communities associated with low-quality plant species should be larger in size than those associated with high-quality plant species. An indication for this pattern might be the observation in a lowland rainforest that chewers with larger body masses, compared to chewers with smaller body masses, consumed less frequently young leaves [71] that provide in average a higher quality than mature leaves. Some authors have found that body mass of phytophagous arthropods is a useful indicator for evaluating the effects of arthropod-induced leaf area loss on ecosystem processes [72,73]. Nonetheless, arthropod abundance is still the preferred proxy to measure the impact of herbivorous arthropods on ecosystems [53,72,74]. Many studies investigating the relation between the abundance of herbivorous arthropods and palatability in forest ecosystems focused on temperate and boreal forest [e.g., 16,31,73]. However, since herbivorous arthropods are major contributors to the loss of photosynthetically active leaf area in tropical forests [29], investigations of plant-herbivore interactions, also in terms of palatability, are of fundamental importance to understanding the ecology of these forests [75].

In our study, we tried to fill the knowledge gab regarding the relationship between herbivorous arthropods and leaf palatability in tropical forest by analyzing the relationship between the abundance of feeding guilds of herbivorous arthropods in tree canopies of tropical montane forests to food quality. Additionally, at the community level, we examined whether the mean body mass of feeding guilds is also related to food quality and whether the Jarman-Bell principle can explain this relationship. Note that we are focusing on arthropod communities and not specific taxa. We selected two feeding guilds, leaf- and rostrum chewers, and determined the number of individuals and the mean body mass of these guilds living on individual trees in montane rainforests. To analyze if relations between palatability and the abundance as well as the mean body mass of these chewing guilds vary depending on the palatability, we applied two different approaches to estimate palatability. One was a feeding trial to experimentally estimate palatability via leaf area loss, the second was a selection of single leaf traits as representative for palatability. Therefore, we evaluated eight leaf traits (S1 Table, S1 File) known to influence leaf palatability to test (1) the hypothesis that leaf traits positively associated with palatability also affect the abundance of the selected guilds positively and vice versa. We assessed (2) whether, consistent with the Jarman-Bell principle, the mean body mass of chewing arthropods increases with the decreasing quality of consumed leaf material. Thus, our question was whether food quality changes the characteristics of arthropod communities and not only selected taxa of herbivores. Finally, to investigate the validity of the effects of certain leaf traits on chewing feeding guilds (3), we examined whether an experimentally estimated measure of palatability leads to the same pattern observed for the single leaf traits used as proxies of palatability.

## Material and methods

### Study area

The study area is located on the eastern slopes of the Andes in southern Ecuador, in the *Reserva Biológica San Francisco* (3°58’30”S 79°4’25”W; RBSF) and the *Parque Nacional Podocarpus* (4°17’0”S 79°0’0”W, PNP) comprising old-growth forests. The area was divided into two study sites, one at ~ 1000 m (PNP) and one ~ 2000 m a.s.l. (RBSF) [76]. Among the tree species used in this study, tree composition exhibited a full species turnover between the elevations [77] (S2 Table). The annual temperature was 19.9°C at 1000 m (precipitation 1825 mm y^-1^) and 14.6°C at 2000 m a.s.l. (precipitation 2007 mm y^-1^) [74]. Within each site, three 100 m × 100 m plots were established. Our study is part of a larger research program (RESPECT: https://vhrz669.hrz.uni-marburg.de/tmf_respect/) and sampling design is a compromise between researchers from very different disciplines [76]. Furthermore, it was originally planned to sample also plots at 3000 m, but the COVID-19 pandemic prevented to collect samples at this elevation level.

### Leaf trait measurements and -selection

During a joined field campaign in February and March 2019, morphological leaf traits, foliar nutrient concentrations, and spectral leaf traits (S3 Table) of 332 trees belonging to 40 tree species were measured (S2 Table). These species were among the most abundant in the plots and varied widely in their life strategies as measured by two functional traits, SLA and wood-specific gravity [77]. At 1000 m a.s.l., 156 trees from 16 species and at 2000 m a.s.l. 176 trees from 15 species were selected. Only trees with a stem diameter at breast height (DBH) ≥ 10 cm were sampled during our study. We collected two sun-exposed branches from each of eight (± one) trees of each sampled species (S2 Table). From each individual 20 representative, mature leaves were selected to measure a variety of leaf traits (S3 Table). For detailed descriptions of techniques measuring traits conventionally in the field and the laboratory see [77]. Additionally, see [78] for detailed descriptions of leaf trait estimated with a spectrometer. After selection of a subset of traits out of the 48 measured variables (for a detailed description of the selection of variables see S1 File), we kept the following eight variables for our analyses characterizing leaf quality (S1 Table): 1^st^ derivates of lignin and cadmium spectral reflectances (as proxies for foliar lignin and cadmium concentrations), specific leaf area, and foliar nitrogen-, phosphorus-, potassium-, calcium-, and iron- concentrations.

### Experimentally estimated palatability

We conducted a non-choice feeding trial with a generalist insect herbivore to experimentally estimate leaf palatability. For the experiment, we chose 316 tree individuals belonging to 41 tree species from the same tree individual pool used for the leaf trait analyses (S2 Table). Per tree species, two to three mature leaves of each branch sample of eight trees were chosen for the experiment. Two leaf disks with a diameter of 20 mm were punched out of each leaf using a steel puncher. In the case of leaves smaller than 20 mm in diameter, leaf parts were punched out until a 20 mm diametrical circle area was reached. Nymphs of the house cricket *Gryllus assimilis* (Fabricius, 1775; Orthoptera, Gryllidae), a non-native generalist chewing herbivore, served as the test organism. Crickets were kept in the laboratory for one week and fed with lettuce and oat flakes. Prior to the experiment, all individuals were isolated and starved for 24 h in small, closed, transparent plastic containers. Afterwards, two replicates (standard deviation ± 0.5) of each one fresh and weighed leaf disk per tree individual together with one isolated cricket were placed for 24 h in a plastic container at an undisturbed site without direct sunlight. Each cricket was used once for the feeding trial, resulting in 708 crickets in total. Around 2% of the crickets did not survive the feeding trial. Four controls per tree species were established, each consisting of one leaf disk in a closed plastic container without the herbivore. After 24 h, each leaf disk was weighed again, stored in a paper bag, and dried in a drying oven at 70°C for at least 72 h. Thereafter, each disk was weighed again to determine its dry mass. To calculate the palatability, we first calculated the water content [%] of a disk as portion of the non-dried weight of a leaf disk after 24 h. To achieve that, we divided the deviation between non-dried weight after 24 h and dry weight after 24 h by the non-dried weight after 24 h and multiplied this value with 100. Then we calculated the expected dry weight of a leaf disk before the 24 h running experiment. To do so, we first multiplied the relative leaf water content with the non-dried weight before 24 h and then subtracted this value from the non-dried weight before 24 h. Afterwards, we calculated the expected dry weight of the leaf area consumed during the experiment by subtracting the dry weight of a disk after 24 h by the above calculated dry weight of a leaf disk before 24 h. To control for leaf water loss via evaporation during the experiment, we calculated the deviation between the above calculated dry weight of a disk before 24 h and the dry weight of a disk after 24 h of disks of the control sets, meaning without a cricket. The leaf water loss was then averaged for each tree species and its standard deviation (sd) was calculated. To identify those dry loss values, which differ significantly from the dry loss via evaporation, we applied a one tailed t-test to create a benchmark and added 1.65 multiplied with the sd of the mean leaf water loss of the control disks to the mean leaf water loss of the control disks. If the calculated expected dry weight of the consumed leaf area was greater than the benchmark, we considered this deviation as consumed dry weight [g]. Finally, we calculated the mean of the consumed dry weight [g] per tree individual and named it in the following palatability [g].

### Arthropod sampling

To collect arthropods in tree canopies, we fogged the canopies of three (± one) trees from 37 tree species of the same pool as for the leaf trait measurements resulting in 133 fogged tree canopies (S2 Table). Fogging was conducted during the field campaigns at ~ 2000 m a.s.l. and ~ 1000 m a.s.l. from September to December in 2018 and 2019, respectively (S2 File). We fogged in the morning, when thermal conditions were adequate, meaning no wind but airflows running from the ground straight up to the canopies. An IGEBA Thermal Fog Generator TF34 E was used with a pyrethroid insecticide mixture consisting of 100 ml of AquaPy^®^ (Bayer Environmental Science), 200 ml of 1, 2-propanediol, 100 ml of glycerine, and 600 ml of H_2_O per liter. Each tree canopy was fogged for five to ten minutes. Beneath each fogged tree canopy, five 1 m × 1 m plastic sheet funnel traps were installed. The traps roughly covered the whole canopy area and allowed the sampling of paralyzed arthropods that fell from the canopy. After three hours of residence time, the arthropods on the funnel traps were brushed out and stored in whirl packs with 70% ethanol.

Due to the large number of arthropod individuals in the samples, they were not sorted taxonomically, but into the following feeding guilds: leaf chewers, rostrum chewers, nectar suckers, sap suckers, predators, and saprobes (S4 Table). In each subsample, the arthropods were counted, dried at 50°C for 24 h in a drying oven, and weighed. The mean body mass of each feeding guild per host tree was then calculated by dividing the dry mass by the number of individuals within the subsample. In the analysis presented herein, we used only leaf chewers and rostrum chewers.

### Data analysis

For the following analyses, we chose only tree individuals within our data set for which we had data for leaf traits, palatability, and both arthropod abundance and mean body mass. Thus, we worked with a subset of 90 tree individuals belonging to 31 species. Hereof, 43 trees from 16 species were located at 1000 m a.s.l. and 47 trees from 15 species were located at 2000 m a.s.l. Their DBH ranged from 10 cm to 68 cm (mean: 16 cm ± 7 cm).

The statistical analysis was done in R version 4.0.2 [79]. Single missing trait values of tree individuals were replaced by mean values from the same tree species (for palatability 13%, lignin 12%, K 2%, and N 1% were replaced with mean values). To achieve approximate normality, the mean body mass, DBH, and all leaf trait variables were log_10_-transformed, except SLA, as well as 1^st^ derivates of single band reflectance at 1420 (D1420), and 1240 nm (D1240). Because the variable ‘palatability’ contained zero values, 0.00001 [g] was added to each value before the log_10_-transformation.

We used linear mixed-effects models to analyze the relation between leaf traits or palatability and chewing arthropods. All predictor variables except ‘palatability’ were z-transformed due to the very different scales of these variables. To analyze the relationship between the abundances of leaf- and rostrum chewers and leaf traits, we constructed generalized linear mixed models (GLMMs) for each guild. Untransformed arthropod counts served as the response variable and z-transformed leaf traits as predictor variables. We, additionally, included the z-transformed DBH as a covariate, as it is positively related to above-ground tree biomass and thus to canopy size [80,81]. We fitted the response variable using a Poisson distribution with the canonical log link. The variable describing at which elevation the tree occurred was included as a fixed (‘site’) effect. In addition, ‘plot’, which specified one of the six 100 m x 100 m plots where the trees were located was added as random effect to the model to account for repeated measurements. Further, tree phylogeny, represented by species nested within genus, nested within family, were included as a random effect. To define the minimal adequate models, we excluded non-significant predictor variables, by applying automated stepwise backward selections, based on models, which converged with as many independent variables as possible. During the reduction process, we forced the random effects to be kept in the models. Both minimal adequate and maximum models were calculated using the R package *buildmer* [82], using the numerical optimization algorithm ‘bound optimization by quadratic approximation’. To control for overdispersion, we used the gof function of the R package *aods3* [83] to calculate the residual degree of freedom (D) and the sum of squared Pearson residuals (X^2^). If the X^2^/D ratio is close to one, no overdispersion occurred. To analyze the relation between both leaf- and rostrum chewer abundance and the experimentally estimated palatability, we implemented again GLMMs using *lme4* [84], by including ‘plot’, to account for pseudo replications, as well as species nested within genus, nested within family as random effect. To control for under- or overdispersion, we used the gof function of the R package *aods3* [83]. To assess the impact of each significant predictor variable on the response variable, we used the *effects* package to plot the partial residuals [85,86].

For analyzing the relation between mean body mass and leaf traits, we constructed linear mixed-effects models (LMEs) for each feeding guild, using the R package *buildmer* [82] and z-transformed leaf traits, including DBH as predictor variables. The response variable mean body mass was log_10_-transformed, and the variable determining at which elevation the tree occurred was included as a fixed (‘site’) effect. The structure of random effects within the model as well as all subsequent analyses were the same as described for the analysis of the abundance data. But, controlling for overdispersion was not necessary. To analyze the relation between chewer mean body mass per feeding guild and the experimentally estimated palatability, we implemented new LMEs using *lmerTest* [87], by again including ‘plot’, to account for pseudoreplications, as well as species nested within genus, nested within family as random effects.

## Results

Among the tree individuals which have been tested for their palatability in the feeding trial, 70% of the trees did not show any dry weight loss greater than the loss caused by evaporation. Thus, the test species did consume little or even no plant material. In contrast, an individual of *Graffenrieda emarginata* (Melastomataceae) possessed with 13.4 mg the highest palatability, followed by trees of *Warszewiczia coccinea* (Rubiaceae, 7.5 mg) and *Clarisia racemosa* (Moraceae, 4.3 mg). A tree of the species *Podocarpus oleifolius* (Podocarpaceae) showed with 0.2 mg the lowest measured palatability value.

When analyzing the correlation between the experimentally estimated palatability and the pre-selected measured leaf traits under the consideration of the elevation level (model 2) Ca, K, Fe, N, and D1240 (cadmium) were significantly correlated with palatability (S5 Table). Evaluating the relation between palatability and leaf traits without considering the effect of the elevation (model 1), the traits Ca, Fe, N, D1240 (cadmium), SLA, and D1420 (lignin) were significantly correlated with palatability (S5 Table).

Regarding the canopy arthropods, chewing arthropods formed the most abundant guild among all collected herbivorous arthropods, with leaf chewers dominating over rostrum chewers (Table 1). Nonetheless, the abundance of chewers per host tree varied by a factor of 140 (Table 2). Based on biomass, chewing arthropods, mainly leaf chewers, were the dominant group (Table 1). Mean body mass between trees varied by a factor of 174 for leaf chewers and 24 for rostrum chewers (Table 2).

**Table 1:** Total number and biomass, either raw values or as a percent, of chewing and sucking arthropods.

	leaf chewers	rostrum chewers	chewer sum	sap sucking herbivores	nectar sucking herbivores	herbivores sum
number of individuals [counts]	19,671	2,997	22,668	11,373	14,157	48,331
number of individuals [%]	40.7	6.2	46.9	23.6	29.5	
weight of all individuals [g]	81.80	7.65	89.45	13.46	5.32	108.24
weight of all individuals [%]	75.6	7.0	82.6	12.4	4.9	

Herbivorous arthropods include, besides chewing feeding guilds, the feeding guilds sap- and nectar suckers. Data were collected and summed up from 90 sampled tree canopies growing at 1000 and 2000 m a.s.l.

**Table 2:** Number of arthropod individuals and biomass on a host tree and mean body mass of an arthropod individual calculated across host trees for the two chewing feeding guilds.

feeding guild	abundance			biomass [g]			mean body mass [mg]		
	min	Mean ± SD	max	min	Mean ± SD	max	min	Mean ± SD	max
**leaf chewers**	8	216 ± 123	697	0.087	0.90 ± 0.65	3.5	0.42	5.3 ± 9.0	86
**rostrum chewers**	5	33 ± 22	120	0.0048	0.085 ± 0.080	0.54	0.38	3.0 ± 2.2	11
**sum chewers**	5	125 ± 127	697	0.0048	0.49 ± 0.62	3.5	0.38	4.1 ± 6.7	87
**sum suckers**	14	141 ± 184	2326	0.0033	0.10 ± 0.10	0.62	0.07	0.90 ± 0.75	4.5

For a comparison, the abundance, biomass, and mean body mass of arthropods on a host tree belonging to sucking feeding guilds (sap- and nectar suckers) are added to the table.

**Fig 1:**
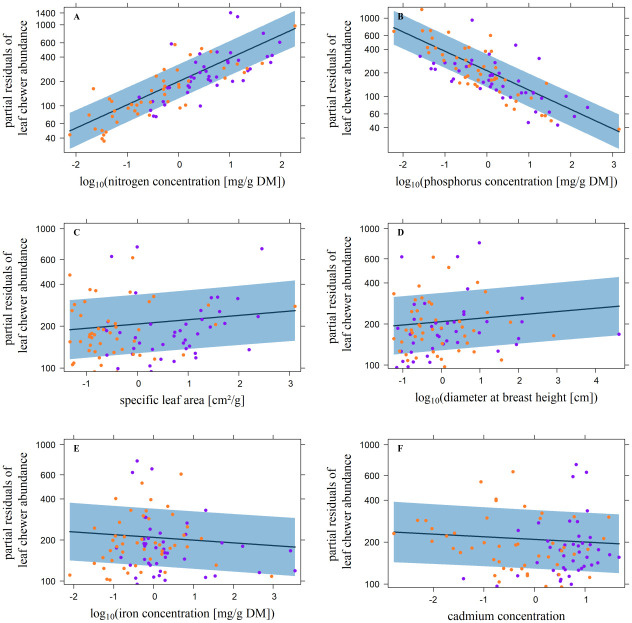
Significant influence of leaf traits on the abundance of leaf chewers. Plotted are the effects of **A**) the log_10_-transformed foliar nitrogen concentrations [mg/g DM], **B**) the log_10_-transformed foliar phosphorus concentrations [mg/g DM], **C**) the specific leaf area [cm^2^/g], **D**) the log_10_-transformed diameter at breast height [cm], **E**) the log_10_-transformed foliar iron concentration [mg/g DM], and **F**) the spectrally measured cadmium concentrations, against the partial residuals of the abundance of leaf chewing arthropods per host tree. Violet and orange dots represent trees at 1000 m and 2000 m a.s.l., respectively. The dark blue lines represent the partial slopes of the effects, and the blue bands define the 95% confidence interval. All predictor variables are z-transformed.

**Fig 2:**
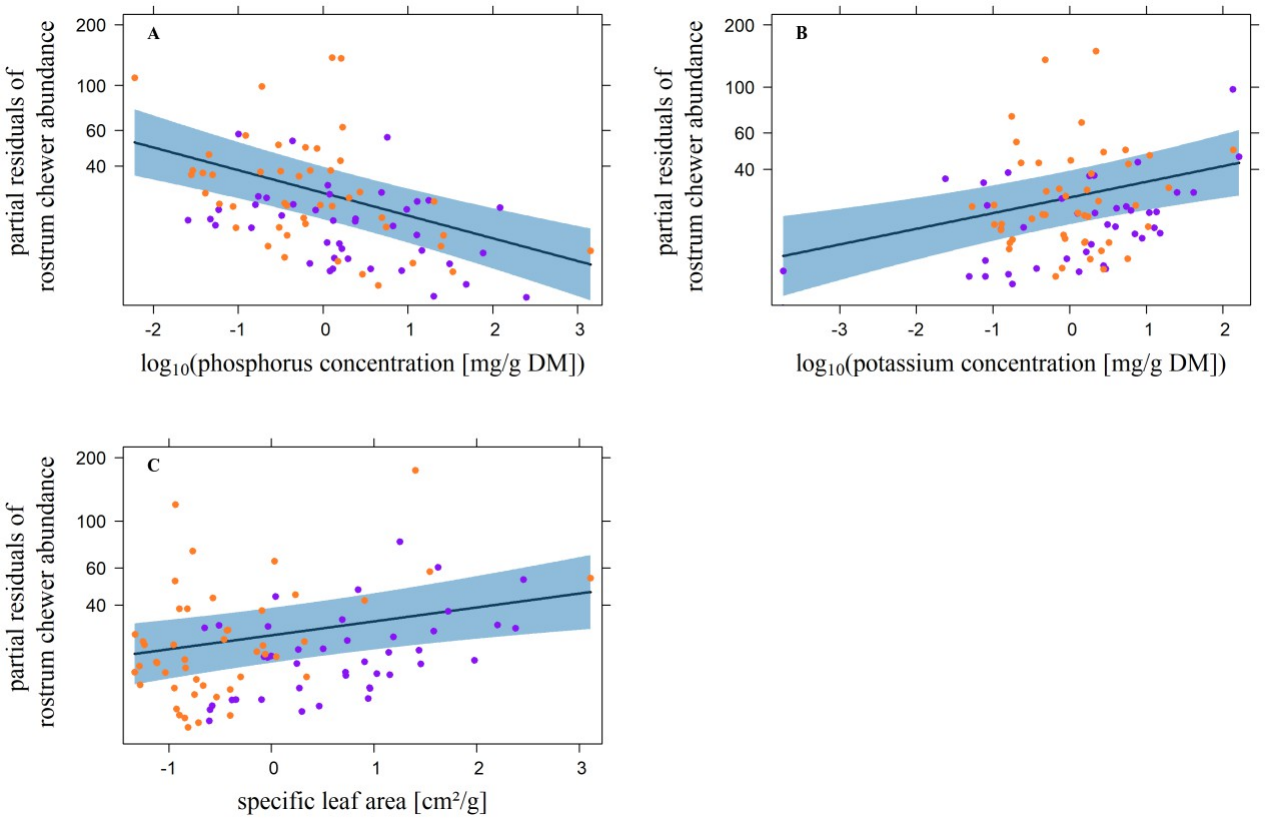
Significant influence of leaf traits on the abundance of rostrum chewers. Plotted are the effects of **A**) the log_10_-transformed foliar phosphorus concentrations [mg/g DM], **B**) the log_10_-transformed foliar potassium concentrations [mg/g DM], and **C**) the specific leaf area [cm^2^/g] against the partial residuals of the abundance of rostrum chewing arthropods per host tree. DM = dry mass. Violet and orange dots represent trees at 1000 m and 2000 m a.s.l., respectively. The dark blue lines represent the partial slopes of the effects, and the blue bands define the 95% confidence interval. All predictor variables are z-transformed.

**Fig 3:**
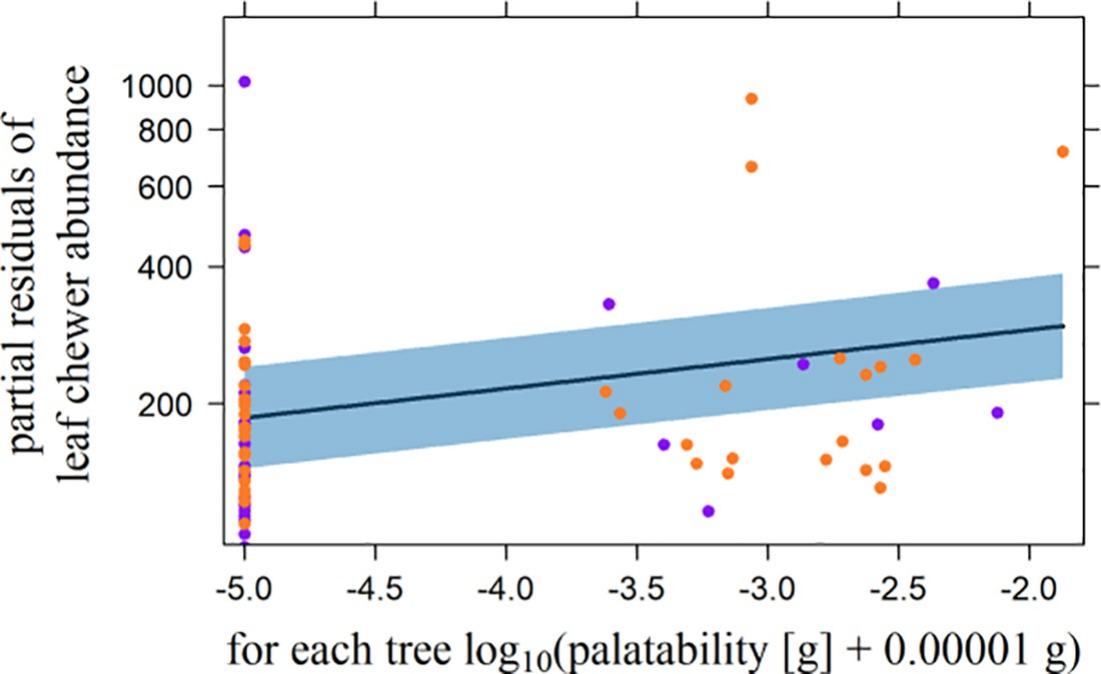
Influence of palatability on leaf chewer abundance per host tree. Palatability [g] was experimentally measured in a feeding trial, with 0.00001 g added to all values before their log_10_ transformation. Plotted is the effect of palatability on the partial residuals of leaf chewer abundance. Violet and orange dots represent trees at 1000 m and 2000 m a.s.l., respectively. The dark blue line represents the partial slope of the effect; the blue band defines the 95% confidence interval.

According to the minimal adequate models for leaf chewers, abundance was positively related to the N concentration, SLA, and DBH. The relationship between the P-, Fe-, and Cd (D1240) concentrations and leaf chewer abundance was negative (Fig 1; Table 3). The data were not overdispersed (X^2^/D = 1467.3/ 1569.9 = 0.9). Rostrum chewer abundance was positively related to SLA, and the K concentration and negatively related to the P concentration (Fig 2; Table 3). Again, there was no overdispersion (X^2^/D = 440.1/465.0 = 0.9). The relationship between palatability and leaf chewer abundance was positive, but there was no significant relationship with rostrum chewer abundance (Fig 3; Table 3). In both cases there was no overdispersion (leaf chewers: X^2^/D = 1949.7/2162.8 = 0.9, rostrum chewers: X^2^/D = 476.3/501.7 = 0.9).

**Table 3:** GLMM models showing the effects of leaf traits on chewer abundance.

model	fixed effects	estimate	SE	p-value
**GLMMs: abundance vs. leaf traits**				
**leaf chewers**				
abundance ~ D1240 + log_10_(DBH) + log_10_(Fe) + log_10_(N) + SLA + log_10_(P) + (1 | family:genus:Species) + (1 | plot)	intercept	5.316	0.247	< 0.001
	D1240	-0.044	0.019	0.0237
	log_10_(DBH)	0.057	0.0107	< 0.001
	log_10_(Fe)	-0.046	0.0114	< 0.001
	SLA	0.071	0.0212	< 0.001
	log_10_(N)	0.662	0.0367	< 0.001
	log_10_(P)	-0.569	0.0247	< 0.001
	**random effects**	**variance**	**SD**	
	family:genus:species (intercept)	0.281	0.531	
	plot (intercept)	0.310	0.557	
**rostrum chewers**				
abundance ~ log_10_(P) + log_10_ (K) + SLA + (1 | family:genus:species) + (1 | family:genus) + (1|plot)	intercept	3.378	0.154	< 0.001
	log_10_(P)	-0.260	0.0489	< 0.001
	log_10_ (K)	0.178	0.0475	< 0.001
	SLA	0.150	0.049	0.00205
	**random effects**	**variance**	**SD**	
	family:genus:species (intercept)	0.137	0.370	
	family:genus (intercept)	0.0279	0.167	
	plot (intercept)	0.0993	0.315	
**GLMMs: abundance vs. palatability**				
**leaf chewers**				
abundance ~ log_10_(palatability + 0.00001) + (1 | plot) + (1 | family)	intercept	5.810	0.157	< 0.001
	log_10_(palatability + 0.00001)	0.116	0.012	< 0.001
	**random effects**	**variance**	**SD**	
	family (intercept)	0.160	0.400	
	plot (intercept)	0.087	0.295	
**rostrum chewers**				
abundance ~ log_10_(palatability + 0.00001) + (1 | plot) + (1 | family:species)	intercept	3.392	0.208	< 0.001
	log_10_(palatability + 0.00001)	0.0105	0.037	0.776
	**random effects**	**variance**	**SD**	
	family:species (intercept)	0.218	0.467	
	plot (intercept)	0.062	0.248	

Minimal adequate model results of the GLMMs of chewer abundance per feeding guild and leaf traits as well as per chewer abundance and experimentally estimated palatability [g]. SE = standard error, SD = standard deviation, log_10_(x) = base 10 logarithmized. Significance was defined at a 5% level.

**Fig 4:**
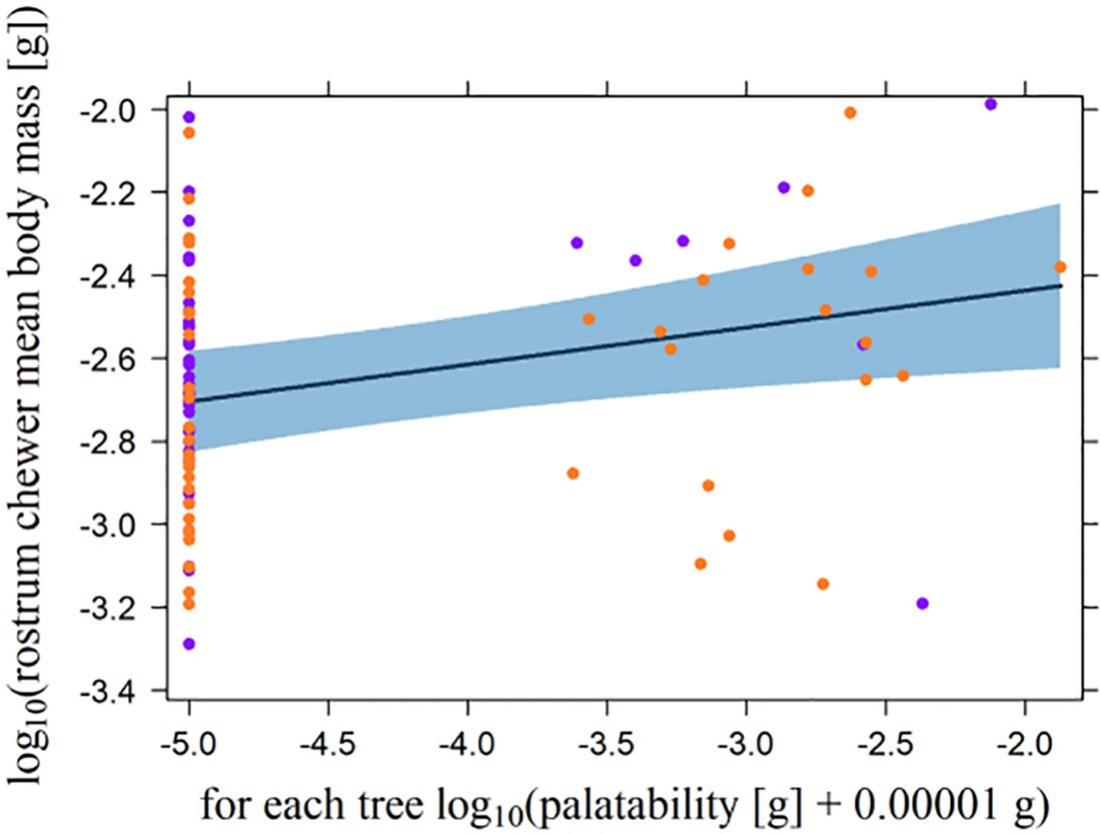
Influence of palatability on rostrum chewer mean body mass per host tree. Palatability [g] was experimentally measured in a feeding trial, with 0.00001 g added to the values before their log_10_ transformation. Plotted is the effect of palatability on the partial residuals of the rostrum chewer mean body mass. Violet and orange dots represent trees at 1000 m and 2000 m a.s.l., respectively. The dark blue line represents the partial slope of the effect; the blue band defines the 95% confidence interval.

Minimal adequate models of the LMEs analyzing the effects of leaf traits on mean body mass did not show any significant effects for neither leaf chewers nor rostrum chewers (Table 4). For leaf chewers, mean body mass was not significantly related to palatability, whereas, for rostrum chewers, the relationship was positive, although this correlation was weak (Fig 4; Table 4).

**Table 4:** LME model results showing the effects of leaf traits on mean body mass of chewers.

minimal adequate model	variables	estimate	SE	p-value
**LMEs: mBM ~ leaf traits**				
**leaf chewers**				
log_10_(mBM) ~ (1 | plot) + (1 | family:genus:species)	intercept	-2.425	0.0391	< 0.001
	**random effects**	**variance**	**SD**	
	species:(genus:family)	0.0	0.0	
	genus:family	0.0	0.0	
	family	0.0	0.0	
	plot	0.00259	0.0509	
**rostrum chewers**				
log_10_(mBM) ~ (1 | plot) + (1 | family:genus:species)	intercept	-2.641	0.0335	< 0.001
	**random effects**	**variance**	**SD**	
	species:(genus:family)	9.72e^-12^	3.12e^-06^	
	genus:family	6.22e^-03^	7.89e^-02^	
	family	0.0	0.0	
	plot	4.63e^-02^	8.01e^-02^	
**LMEs: mBM ~ palatability**				
**leaf chewers**				
log_10_(mBM) ~ log_10_(palatability + 0.00001) + (1 | plot) + (1 | family:genus:species)	intercept	-2.602	0.140	< 0.001
	log_10_(palatability + 0.00001)	-0.0413	0.0312	0.192
	**random effects**	**variance**	**SD**	
	species:(genus:family)	7.76e^-05^	8.91e^-03^	
	genus:family	3.30e^-10^	1.82e^-05^	
	family	0.0	0.0	
	plot	1.08e^-03^	0.292	
**rostrum chewers**				
log_10_(mBM) ~ log_10_(palatability + 0.00001) + (1 | plot) + (1 | family:genus:species)	intercept	-2.258	0.154	< 0.001
	log_10_(palatability + 0.00001)	0.0892	0.0328	0.00795
	**random effects**	**variance**	**SD**	
	species:(genus:family)	0.0	0.0	
	genus:family	0.0	0.0	
	family	0.0	0.0	
	plot	0.0114	0.107	

Minimal adequate model results of the LMEs of mean body mass per feeding guild and leaf traits as well as the LMEs of mean body mass per feeding guild and experimentally estimated palatability [g]. mBM = mean body mass, SE = standard error, SD = standard deviation, log_10_(x) = base 10 logarithmized. Significance was defined at a 5% level.

## Discussion

The dominance of leaf chewers within the arthropod communities of tree canopies is in accordance with earlier studies (Table 1) [26–28]. However, in contrast to the variety of taxonomic groups belonging to the leaf chewing feeding guild (e.g., Orthoptera, Lepidoptera, Coleoptera, Phasmatodea), rostrum chewers represent only one taxon, the Curculionoidea [88]. This difference can partly explain the lower abundance of rostrum chewers. Arthropod abundance between host trees varied strongly (Table 2), which in part might be attributable to differences in the canopy sizes of host plants, as larger canopies can harbor higher arthropod diversities and abundances (species-area relationship) [89,90]. Support for this relationship is the positive effect of DBH on leaf chewer abundance since DBH is as a proxy for above-ground biomass and thus canopy size [80,81].

### Relation between leaf traits and experimentally estimated palatability

The leaf traits which were significantly related to experimentally estimated palatability showed for three (N, K, and Fe) out of eight traits correlations we expected from the literature. N is an essential nutrient for herbivores and its positive effect on palatability was documented several times in previous studies [e.g., 39,43,44]. K in contrast was shown to decrease palatability in different feeding trials [40,48,49], which also coincided with our results. Regarding the metal Fe, we expected and observed a negative relation to palatability in our data since Fe can have fitness reducing effects for herbivores, if it is accumulated in plants [35,41]. However, the remaining four leaf traits and elevation were related to palatability contrary to our expectations. SLA was negatively related to palatability, whereby we assumed an increase in palatability with increasing SLA since leaves with higher SLAs tend to have larger and thinner leaves, higher growth rates and invest less into plant defense than leaves with smaller SLAs [44]. Nonetheless, [91] showed that the hemipteran *Tingis tecomae*, provided with standardized leaf disks taken from leaves of different sizes did not prefer leaf disks from larger leaves in a feeding trial, although *T*. *tecomae* prefers larger leaves in nature. In contrast, the hemipteran *Rhabdotalebra* chose leaf disks from larger leaves in the feeding trial as it chose larger leaves in nature. This observation demonstrates, that making clear assumptions regarding the SLA-palatability relationship is difficult. Thus, our observed negative relation between SLA and palatability should be taken with caution, especially when considering the positive relation between both leaf- and rostrum chewer abundance in our study. Further, the positive correlation between the secondary metabolite lignin (D1420) and palatability was contrary to our expectation of a negative relation, which was detected in many other studies [e.g., 33,38,41,51,52]. However, this result was consistent with the negative relationship of SLA with palatability. Furthermore, positive relations can occur [92] indicating that explicit predictions are difficult to make. This ambiguity was supported by the non-expected negative relation between Ca and palatability. Overall, the relations between leaf traits and palatability observed in this study in comparison to observations described in literature indicate that there tend to be single traits like N and SLA, which seemed to be more important for herbivorous arthropods than other traits like secondary metabolites such as lignin [93]. Finally, increasing elevation can have both negative and positive effects on palatability [e.g., 37,44,93,94]. This relies on changing characteristics of leaf traits with elevation. In our study, the concentrations of all leaf traits significantly influencing palatability (Ca, D1240, Fe, K, and N) decreased with increasing elevation. Thus, elevation effects should always be interpreted in combination with effects of leaf traits.

### Chewer abundance

Chewer abundance was affected by several leaf traits (Figs 1 and 2; Table 3). SLA had positive and the P concentration negative effects on leaf- and rostrum chewers. The effects of the remaining leaf traits were guild-specific. However, SLA and N, leaf traits often assumed to increase palatability, positively affected chewer abundance, as expected from reports in the literature [36,39,42,95,96]. While the foliar N concentration was the main driver of leaf chewer abundance, it had no effect on rostrum chewer abundance in our study. To our knowledge, this observation has not been made before.

Negative effects were expected at foliar P concentrations > 1%, i.e., far higher than the global average determined in terrestrial foliage [20,37]. Nonetheless, negative effects were found at lower concentrations (Figs 1B and 2A), presumably due to the reduction in herbivore fitness caused by nutrient concentrations outside consumer-specific requirements [21]. This may have been the case among generalist herbivores, which account for a significant portion of chewing insects [96,97]. In contrast to monophagous herbivores, polyphagous feeding generalists might be unable to adapt to the nutrient concentrations of the host plant they temporarily feed on. Further, the positive relation between the K concentration and rostrum chewer abundance (Fig 2B) was contrary to our expectation, since changes of K concentrations in plants were shown to possibly reduce the growth rate and the survivorship of herbivores [40]. Thus, we expected a negative relation to chewer abundance. However, in accordance with our observation, a positive relation between the consumption rate of the rostrum chewer *Otiorhynchus sulcatus* (Curculionidae) and foliar potassium concentration of blackcurrant is documented in literature [98]. The positive relation shown in our study might be due to the importance of K^+^ ions for various physiological and mechanical processes of especially insects like neurotransmission, salivary production, reproduction, and development [99]. Regarding the two metals Fe and Cd, which had weak but significant negative influences on only leaf chewer abundances, the results were in accordance with expectations based on literature [35,54,55,100]. According to the elemental defense hypothesis, increasing concentrations of metals reduce the herbivore- and increase the host plant fitness [41], a possible explanation for the negative relation between the Fe and Cd concentrations and leaf chewer abundances. However, effects of Fe on herbivores are little explored [41]. Additionally, [41] compiled several studies demonstrating positive or no effects of Cd on herbivore fitness. This might explain the non-significant influence of the Cd and the Fe concentrations on the rostrum chewer abundance in our study.

The positive effect of the experimentally estimated palatability on leaf chewer abundance was in accordance with our expectation [101] and could be due to the dominating correlation between the N concentration and both palatability and leaf chewer abundance. The effect of the Fe concentration could have supported this pattern. In return, the non-significant correlations between rostrum chewer abundance and both the N and Fe concentrations could explain the unimportance of the experimentally estimated palatability for the abundance of rostrum chewers.

Nonetheless, in our study, we only measured the leaf traits of mature leaves, which accumulate herbivorous damage over time and have trait characteristics differing from those of young leaves [16,29,33]. Since chewing arthropods feed predominately on young leaves [29,33,71,88,102,103], the effects of leaf traits on chewer abundance as determined in our study should be interpreted with caution. Our results indeed demonstrate an influence of leaf traits on chewer abundance. However, which traits are decisive and how they affect chewers depends on the surrounding environmental conditions and is specific to each feeding guild. Furthermore, the diffuse effects of leaf traits on chewing arthropod abundances in this study indicate that, on the community level, plant species-specific characteristics and diet-quality-driven physiological processes might play a minor role in influencing abundance, and factors such as predation and parasitism or parasitoids could be far more critical [104–106]. This assumption is supported by the inconsistent and hardly interpretable patterns observed when analyzing the data per elevation separately (S6 Table).

### Chewer mean body mass

With respect to chewing arthropods, the Jarman-Bell principle was not supported at the community level. For leaf chewers, neither the model results for leaf traits nor experimentally estimated palatability showed a correlation between mean body mass and palatability (Table 4, S7 Table). For rostrum chewers, mean body mass was not correlated with leaf traits (Table 4, S7 Table). However, the experimentally estimated palatability was positively related to rostrum chewer mean body mass. This pattern was contrary to the pattern expected from the Jarman-Bell principle. The result supports the assumption which was suggested by [88] that, unlike leaf chewers which contain a relatively great portion of soft and extensible caterpillars and larva, rostrum chewers which are adults with a little extensible exoskeleton depend on food of higher quality because of their narrower gapes and, in some species, their smaller gut sizes.

Our results indicate that the mean body masses of communities of leaf- and rostrum chewers may not be driven by body mass–energy relationships. Other mechanisms possibly influencing the body mass of chewers could be that the gut size can increase with increasing body mass and as compensation for a low food quality [107]. Further, symbiotic bacteria living in the gut could compensate for a low diet quality [22,108]. Additionally, abiotic factors such as temperature might have effects on body mass (temperature-size rule) [109] superimposing the physiological effects of leaf traits.

### Considerations and constraints

Our results demonstrated that the abundance and mean body mass of chewing arthropod feeding guilds in tree canopies are affected by specific compositions of leaf traits. Biotic interactions, such as predation and parasitism or parasitoids [102,104,105,110,111], as well as abiotic conditions, such as temperature, solar radiation, wind, precipitation, and air pressure [110–113], might also influence chewing arthropod abundance and mean body mass in the canopies of tropical montane rainforests. Consequently, the effects of foliar morphological, nutrient, or defense characteristics would be masked. Furthermore, refining feeding guilds might improve models (e.g., by splitting chewing herbivores into fruit-, seed-, flower-, pollen-, phloem-, xylem-, or bark chewers) [16,26,31]. Additionally, different sampling methods, such as fogging, flight-interception traps, and malaise traps, as well as repeated sampling and sampling during different seasons, would yield collections differing in their arthropod composition [114–117]. For example, among the chewing feeding guilds our collection may have lacked a significant portion of leaf rollers, which probably did not fall when paralyzed by the insecticide after the fogging process. Fast flying arthropods could have also escaped, alarmed by the noisy fogger.

Additionally, our samples were contaminated with arthropods either occupying neighboring tree canopies or feeding on the epiphytes and epiphylls growing on the target canopy [117]. The inclusion of several tourist species was also likely [118]. Furthermore, host specificity might not have been as high as expected [96,117,119], resulting in more generalist herbivores feeding only temporally on the target canopy. These considerations and constraints highlight the difficulties in obtaining differentiated assessments of leaf traits’ effects on chewing arthropods communities in the field. Nonetheless, on a large scale, our study results offer insights into leaf traits-chewers relationships in tree canopies of tropical montane rainforests. In the feeding trial, the use of a non-native generalist insect might have biased the palatability results, as they would not have reflected possible co-evolutive adaptations of the local arthropod assemblage. Additionally, using the calculated dry weight of the consumed leaf disk parts as proxy for palatability by accounting for the evaporation may have led to an underestimation of the consumed dry mass by overrating the evaporation. Further, the comparison between the leaf traits being significantly related to the experimentally estimated palatability and the leaf traits affecting chewer abundance and mean body mass show that using an experimentally estimated palatability measure as proxy for a variety of leaf traits to analyze their effects on chewer herbivores is difficult as traits being related to that palatability measure are not consequently related to chewer abundance or mean body mass and vice versa. Thus, both approaches do not always lead to the same conclusions.

## Conclusion

Nonetheless, on a larger scale, the results of our study offer insights into relations between leaf- and rostrum-chewing arthropods in the tree canopies of tropical montane rainforests and leaf traits of host plants. On a community level, some leaf traits had effects on chewer abundance as we expected based on literature, other not. Furthermore, the results were inconsistent with the Jarman-Bell principle, as the mean body mass of leaf chewers showed significant correlations with neither experimentally estimated palatability nor leaf traits. The mean body mass of rostrum chewers was also not correlated to specific leaf traits, but it correlated contrary to the expectation positively with experimentally estimated palatability. Our results thus demonstrate the complex interplay between the leaf traits of host plants and the herbivore communities of chewing arthropods in tree canopies of tropical montane rainforests. Estimating the importance of the Jarman-Bell principle for certain specific arthropod taxa by determining the influences of plant traits on their arthropod mean body mass, could be a further, future step towards the understanding of diet quality-herbivore relationships. However, it seems, that on a community level, diet quality plays a secondary role in shaping leaf- and rostrum chewer abundance and mean body mass. To better understand if other factors, such as the microclimate, predation or further abiotic or biotic interactions in tree canopies are more important in influencing communities of chewing arthropod herbivores will require further, more refined analyses to disentangle their role in ecosystem processes.

## Supporting information

S1 Fig Correlation matrices of leaf traits. (TIF)

S2 Fig Phylogenetic principal component analysis of the similarity of various leaf traits. (TIF)

S1 Table Selection of studies evaluating the influence of leaf traits used in this study on palatability. (DOCX)

S2 Table Examined tree species with the elevation level of occurrence and the number of replicates. (DOCX)

S3 Table Leaf traits generated in the context of the RESPECT project. (DOCX)

S4 Table Feeding guilds of larval and adult arthropod taxa. (DOCX)

S5 Table Minimal adequate linear models showing the relations between leaf traits and palatability. (DOCX)

S6 Table GLMM models showing the effects of leaf traits on chewer abundance per elevation. (DOCX)

S7 Table LME models showing the effects of leaf traits on mean body mass [g] of chewers per elevation. (DOCX)

S1 Dataset Raw dataset for 90 tree individuals used for the statistical analysis. (CSV)

S2 Dataset Metadata for the S1 Dataset. (XLSX)

S1 File Variable selection. (DOCX)

S2 File Inclusivity in global research. (DOCX)
