# Supplementary figures and images for: Effects of leaf traits of tropical trees on the abundance and body mass of herbivorous arthropod communities
Source: PLoS One. 2023 Nov 7;18(11):e0288276. doi: 10.1371/journal.pone.0288276 (PMC10629635; doi:10.1371/journal.pone.0288276)

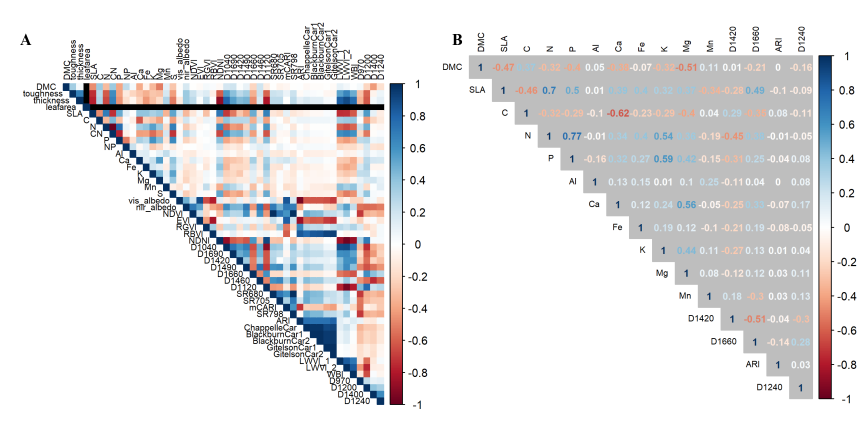

Supplement: S1 Fig — (TIF) [file pone.0288276.s001.tif]

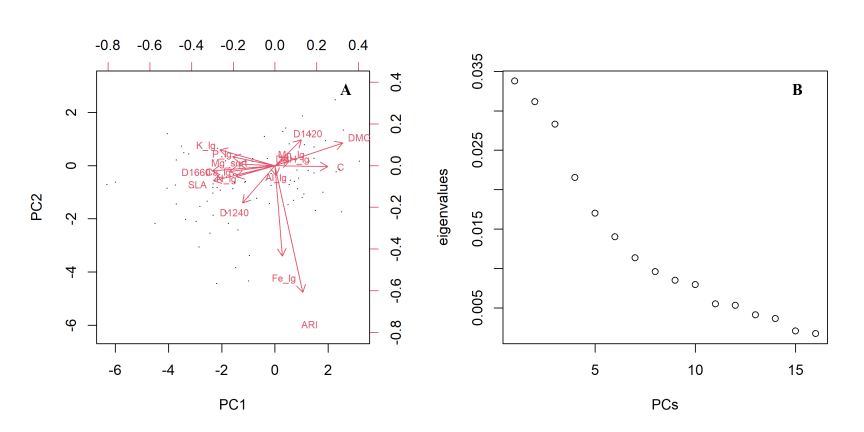

Supplement: S2 Fig — (TIF) [file pone.0288276.s002.tif]
