# Supplementary material for: Effects of leaf traits of tropical trees on the abundance and body mass of herbivorous arthropod communities
Source: PLoS One. 2023 Nov 7;18(11):e0288276. doi: 10.1371/journal.pone.0288276 (PMC10629635; doi:10.1371/journal.pone.0288276)
Supplement: S1 Table — (DOCX) [file pone.0288276.s003.docx]

**S1 Table. Selection of studies evaluating the influence of leaf traits used in this study on palatability.**

| **leaf trait** | **relation to palatability/herbivory** | **references** | **log_10_ transformation** |
| --- | --- | --- | --- |
| **lignin**  (D1420^1^) | - | Coley, 1983;  Coley et al., 1985;  Moreira et al., 2017;  L. Poorter et al., 2004;  Rosenthal & Janzen, 1979 | no |
|  | + | Mason et al., 2011 |  |
| **cadmium**  (D1240^2^) | - | Plaza et al., 2015*;*  *Kazemi‐Dinan et al., 2014;*  *Coleman et al., 2005* | no |
| **iron**  (Fe)  **[mg/g DM]** | - | Ribeiro et al., 2017 | yes |
|  | + | *Awmack & Leather, 2002* |  |
| **potassium**  (K)  **[mg/g DM]** | - | *Armengaud et al., 2010;*  *Stamp, 1994;*  *Stamp & Harmon, 1991* | yes |
|  | hs | *Wulfson & Stamp, 1991* |  |
| **specific leaf area** (SLA) **[cm²/g]** | + | *Pérez-Harguindeguy et al., 2003;*  *Schädler et al., 2003* | no |
|  | 0 | *Descombes et al., 2017* |  |
|  | - | *Dostálek et al., 2020;*  *L.* Poorter et al., 2004 |  |
| **phosphorus**  (P)  **[mg/g DM]** | + | *Khan et al., 2016;*  Perkins et al., 2004;  Werner & Homeier, 2015 | yes |
|  | hs | Cease et al., 2016 |  |
|  | - | Garibaldi et al., 2011 |  |
| **nitrogen**  (N)  **[mg/g DM]** | + | Coley, 1983;  T. Cornelissen & Stiling, 2006;  *Descombes et al., 2017;*  *Kurokawa et al., 2010;*  Mason et al., 2011;  *Pérez-Harguindeguy et al., 2003;*  *Schädler et al., 2003;*  Werner & Homeier, 2015 | yes |
| **calcium**  (Ca)  **[mg/g DM]** | + | Awmack & Leather, 2002*;*  Forbes et al., 2017 | yes |

Selection of studies describing observed effects of leaf traits used in this study on the palatability of leaves and thus their herbivory rates. Shown are example studies to document relations that can be expected. Additionally, variable transformations are listed if needed for the analyses in this study. Studies in italics indicate laboratory feeding experiments that measured palatability; those not in italics were field measurements of herbivory, measured as leaf area loss. DM = dry mass, - = negative relationship, + = positive relationship, hs = hump-shaped relation, 0 = no relation; log_10_ = logarithm of base 10.

^1^: first derivate of reflectance at the spectral wavelength of 1.42 nm as a proxy for foliar lignin concentration (Curran, 1989).

^2^: first derivate of reflectance at the spectral wavelength of 1.24 nm as a proxy for foliar cadmium concentration (Rosso et al., 2005).

**References**

Armengaud, P., Breitling, R., & Amtmann, A. (2010). Coronatine-insensitive 1 (COI1) mediates transcriptional responses of Arabidopsis thaliana to external potassium supply. *Molecular Plant*, *3*(2), 390–405. https://doi.org/10.1093/mp/ssq012

Awmack, C. S., & Leather, S. R. (2002). Host Plant Quality and Fecundity in Herbivorous Insects. *Annual Review of Entomology*, *47*(1), 817–844. papers3://publication/uuid/3CB51458-7BDB-45B5-94FB-754F527A0B4F

Cease, A. J., Fay, M., Elser, J. J., & Harrison, J. F. (2016). Dietary phosphate affects food selection, post-ingestive phosphorus fate, and performance of a polyphagous herbivore. *Journal of Experimental Biology*, *219*(1), 64–72. https://doi.org/10.1242/jeb.126847

Coley, P. D. (1983). Herbivory and Defensive Characteristics of Tree Species in a Lowland Tropical Forest. *Ecological Society of America*, *53*(2), 209–233.

Coley, P. D., Bryant, J. P., & Chapin, F. S. (1985). Resource availability and plant antiherbivore defense. *Science*, *230*(4728), 895–899.

Cornelissen, T., & Stiling, P. (2006). Responses of different herbivore guilds to nutrient addition and natural enemy exclusion. *Ecoscience*, *13*(1), 66–74. https://doi.org/10.2980/1195-6860(2006)13[66:RODHGT]2.0.CO;2

Curran, P. J. (1989). Remote sensing of foliar chemistry. *Remote Sensing of Environment*, *30*(3), 271–278. https://doi.org/10.1016/0034-4257(89)90069-2

Descombes, P., Marchon, J., Pradervand, J. N., Bilat, J., Guisan, A., Rasmann, S., & Pellissier, L. (2017). Community-level plant palatability increases with elevation as insect herbivore abundance declines. *Journal of Ecology*, *105*(1), 142–151. https://doi.org/10.1111/1365-2745.12664

Dostálek, T., Rokaya, M. B., & Münzbergová, Z. (2020). Plant palatability and trait responses to experimental warming. *Scientific Reports*, *10*(1), 1–12.

Forbes, R. J., Watson, S. J., & Steinbauer, M. J. (2017). Multiple plant traits influence community composition of insect herbivores: a comparison of two understorey shrubs. *Arthropod-Plant Interactions*, *11*(6), 889–899. https://doi.org/10.1007/s11829-017-9545-1

Garibaldi, L. A., Kitzberger, T., & Chaneton, E. J. (2011). Environmental and genetic control of insect abundance and herbivory along a forest elevational gradient. *Oecologia*, *167*(1), 117–129.

Kazemi‐Dinan, A. Thomaschky, S., Stein, R. J., Krämer, U., & Müller, C. (2014). Zinc and cadmium hyperaccumulation act as deterrents towards specialist herbivores and impede the performance of a generalist herbivore. *New Phytologist*, *202*(2), 628–639.

Khan, G. A., Vogiatzaki, E., Glauser, G., & Poirier, Y. (2016). Phosphate deficiency induces the jasmonate pathway and enhances resistance to insect herbivory. *Plant Physiology*, *171*(1), 632–644. https://doi.org/10.1104/pp.16.00278

Kurokawa, H., Peltzer, D. A., & Wardle, D. A. (2010). Plant traits, leaf palatability and litter decomposability for co-occurring woody species differing in invasion status and nitrogen fixation ability. *Functional Ecology*, *24*(3), 513–523. https://doi.org/10.1111/j.1365-2435.2009.01676.x

Mason, N. W. H., Carswell, F. E., Richardson, S. J., & Burrows, L. E. (2011). Leaf palatability and decomposability increase during a 200-year-old post-cultural woody succession in New Zealand. *Journal of Vegetation Science*, *22*(1), 6–17. https://doi.org/10.1111/j.1654-1103.2010.01223.x

Moreira, X., Glauser, G., & Abdala-Roberts, L. (2017). Interactive effects of plant neighbourhood and ontogeny on insect herbivory and plant defensive traits. *Scientific Reports*, *7*(1), 1–9. https://doi.org/10.1038/s41598-017-04314-3

Pérez-Harguindeguy, N., Díaz, S., Vendramini, F., Cornelissen, J. H., Gurvich, D. E., & Cabido, M. (2003). Leaf traits and herbivore selection in the field and in cafeteria experiments. *Austral Ecology*, *28*(6), 642–650.

Perkins, M. C., Woods, H. A., Harrison, J. F., & Elser, J. J. (2004). Dietary phosphorus affects the growth of larval Manduca sexta. *Archives of Insect Biochemistry and Physiology*, *55*(3), 153–168. https://doi.org/10.1002/arch.10133

Plaza, S., Weber, J., Pajonk, S., Thomas, J., Talke, I. N., Schellenberg, M., Pradervand, S., Burla, B., Geisler, M., Martinoia, E., & Krämer, U. (2015). Wounding of Arabidopsis halleri leaves enhances cadmium accumulation that acts as a defense against herbivory. *BioMetals*, *28*(3), 521–528. https://doi.org/10.1007/s10534-015-9829-9

Poorter, L., Van De Plassche, M., Willems, S., & Boot, R. G. A. (2004). Leaf traits and herbivory rates of tropical tree species differing in successional status. *Plant Biology*, *6*(6), 746–754. https://doi.org/10.1055/s-2004-821269

Ribeiro, S. P., Londe, V., Bueno, A. P., Barbosa, J. S., Corrêa, T. L., Soeltl, T., Maia, M., Pinto, V. D., de França Dueli, G., de Sousa, H. C., Kozovits, A. R., & Nalini, H. A. (2017). Plant defense against leaf herbivory based on metal accumulation: examples from a tropical high altitude ecosystem. *Plant Species Biology*, *32*(2), 147–155. https://doi.org/10.1111/1442-1984.12136

Rosenthal, G. A., & Janzen, D. H. (1979). *Herbivores their interaction with secondary plant metabolites*.

Rosso, P. H., Pushnik, J. C., Lay, M., & Ustin, S. L. (2005). Reflectance properties and physiological responses of Salicornia virginica to heavy metal and petroleum contamination. *Environmental Pollution*, *137*(2), 241–252. https://doi.org/10.1016/j.envpol.2005.02.025

Schädler, M., Jung, G., Auge, H., & Brandl, R. (2003). Palatability, decomposition and insect herbivory: Patterns in a successional old-field plant community. *Oikos*, *103*(1), 121–132. https://doi.org/10.1034/j.1600-0706.2003.12659.x

Stamp, N. E. (1994). Simultaneous effects of potassium, rutin and temperature on performance of Manduca sexta caterpillars. *Entomologia Experimentalis et Applicata*, *72*(2), 135–143. https://doi.org/10.1111/j.1570-7458.1994.tb01811.x

Stamp, N. E., & Harmon, G. D. (1991). Effect of potassium and sodium on fecundity and survivorship of Japanese beetles. *Oikos*, 299–305.

Werner, F. A., & Homeier, J. (2015). Is tropical montane forest heterogeneity promoted by a resource-driven feedback cycle? Evidence from nutrient relations, herbivory and litter decomposition along a topographical gradient. *Functional Ecology*, *29*(3), 430–440. https://doi.org/10.1111/1365-2435.12351

Wulfson, L. S., & Stamp, N. E. (1991). Effects of potassium levels over the range occuring in host plants on the growth of an insect herbivore (Manduca sexto). *Entomologia Experimentalis et Applicata*, *61*(1), 59–72.
