# Supplementary material for: Effects of leaf traits of tropical trees on the abundance and body mass of herbivorous arthropod communities
Source: PLoS One. 2023 Nov 7;18(11):e0288276. doi: 10.1371/journal.pone.0288276 (PMC10629635; doi:10.1371/journal.pone.0288276)
Supplement: S2 Table — (DOCX) [file pone.0288276.s004.docx]

**S2 Table.** **Examined tree species with the elevation level of occurrence and the number of replicates.**

| **species** | **family** | **elevation [m a.s.l.]** | **replicates for leaf trait estimation** | **replicates for palatability experiment** | **replicates for arthropod collection** | **replicates used in the analyses** |
| --- | --- | --- | --- | --- | --- | --- |
| *Alchornea glandulosa* | Euphorbiaceae | 1000 | 9 | 8 | 4 | 0 |
| ***Alchornea lojaensis*** | Euphorbiaceae | 2000 | 9 | 12 | 5 | 3 |
| ***Alzatea verticillata*** | Alzateaceae | 2000 | 9 | 8 | 4 | 4 |
| ***Aspidosperma rigidum*** | Apocynaceae | 1000 | 8 | 8 | 6 | 1 |
| *Cecropia angustifolia* | Urticaceae | 2000 | 10 | 10 | 0 | 0 |
| ***Chrysochlamys membranacea*** | Clusiaceae | 1000 | 8 | 9 | 3 | 3 |
| ***Clarisia racemosa*** | Moraceae | 1000 | 8 | 9 | 4 | 3 |
| *Clusia ducuoides* | Clusiaceae | 2000 | 12 | 13 | 4 | 0 |
| *Elaeagia mollis* | Rubiaceae | 2000 | 8 | 8 | 0 | 0 |
| ***Graffenrieda emarginata*** | Melastomataceae | 2000 | 9 | 10 | 4 | 3 |
| ***Grias peruviana*** | Lecythidaceae | 1000 | 8 | 8 | 5 | 4 |
| ***Guarea macrophylla*** | Meliaceae | 1000 | 8 | 8 | 4 | 4 |
| ***Guatteria pastazae*** | Annonaceae | 1000 | 6 | 6 | 4 | 2 |
| ***Hedyosmum* cf *goudotianum*** | Chloranthaceae | 2000 | 8 | 9 | 5 | 5 |
| ***Heliocarpus americanus*** | Malvaceae | 2000 | 9 | 9 | 1 | 1 |
| ***Hieronyma fendleri*** | Phyllanthaceae | 2000 | 9 | 12 | 4 | 4 |
| ***Hieronyma oblonga*** | Phyllanthaceae | 1000 | 8 | 8 | 4 | 4 |
| ***Ladenbergia oblongifolia*** | Rubiaceae | 1000 | 8 | 8 | 4 | 1 |
| ***Leonia crassa*** | Violaceae | 1000 | 8 | 8 | 5 | 1 |
| ***Matayba inelegans*** | Sapindaceae | 2000 | 11 | 10 | 6 | 5 |
| *Meriania franciscana* | Melastomataceae | 2000 | 6 | 6 | 0 | 0 |
| ***Miconia* aff *punctata*** | Melastomataceae | 1000 | 8 | 8 | 4 | 2 |
| ***Miconia calophylla*** | Melastomataceae | 2000 | 8 | 10 | 4 | 4 |
| ***Mollia gracilis*** | Malvaceae | 1000 | 7 | 8 | 3 | 2 |
| ***Myrcia spnov*** | Myrtaceae | 2000 | 8 | 13 | 4 | 4 |
| ***Myrsine coriacea*** | Primulaceae | 2000 | 8 | 11 | 3 | 3 |
| ***Naucleopsis francisci*** | Moraceae | 2000 | 9 | 12 | 2 | 2 |
| ***Ocotea andina*** | Lauraceae | 2000 | 9 | 9 | 3 | 2 |
| ***Ocotea* JH6030** | Lauraceae | 1000 | 8 | 7 | 4 | 3 |
| *Otoba parvifolia* | Myristicaceae | 1000 | 8 | 8 | 4 | 0 |
| *Parkia* sp.1 | Fabaceae | 1000 | 8 | 5 | 0 | 0 |
| ***Podocarpus oleifolius*** | Podocarpaceae | 2000 | 9 | 11 | 4 | 4 |
| *Pourouma cecropiifolia* | Urticaceae | 1000 | 6 | 4 | 0 | 0 |
| ***Pouteria torta*** | Sapotaceae | 1000 | 8 | 7 | 4 | 2 |
| ***Pseudolmedia laevis*** | Moraceae | 1000 | 8 | 8 | 3 | 3 |
| ***Ruagea glabra*** | Meliaceae | 2000 | 8 | 11 | 2 | 1 |
| ***Saurauia* JH5994** | Actinidiaceae | 1000 | 8 | 8 | 5 | 5 |
| ***Tapirira guianensis*** | Anacardiaceae | 2000 | 9 | 8 | 2 | 2 |
| ***Warszewiczia coccinea*** | Rubiaceae | 1000 | 8 | 8 | 5 | 3 |
| *Weinmannia microphylla* | Cunoniaceae | 2000 | 8 | 12 | 0 | 0 |
| NA | NA | 1000 | 0 | 0 | 1 | 0 |

Listed are the number of replicates of species used for the leaf trait estimation, the feeding trial, the arthropod collection, and finally the number of replicates used for the analyses in this study. Species included in the analyses are in bold.
