# Supplementary material for: Effects of leaf traits of tropical trees on the abundance and body mass of herbivorous arthropod communities
Source: PLoS One. 2023 Nov 7;18(11):e0288276. doi: 10.1371/journal.pone.0288276 (PMC10629635; doi:10.1371/journal.pone.0288276)
Supplement: S3 Table — (DOCX) [file pone.0288276.s005.docx]

**S3 Table. Leaf traits generated in the context of the RESPECT project.**

| **spectrally measured traits** | **conventionally measured traits** |
| --- | --- |
| visible incoming radiation (vis albedo) | leaf dry matter content (DMC) |
| near-infrared incoming radiation (nir albedo) | leaf toughness |
| normalized difference vegetation index (NDVI) | leaf thickness |
| enhanced vegetation index (EVI) | leaf area |
| red green vegetation index (RGVI) | **specific leaf area (SLA)** |
| red blue vegetation index (RBVI) | leaf carbon content (C) |
| normalized difference nitrogen index (NDNI) | **leaf nitrogen content (N)** |
| oil (D1040) [1] | **leaf phosphorus content (P)** |
| lignin, starch, protein, nitrogen (D1690) [1] | leaf N:P ratio |
| **lignin (D1420)** [1] | leaf aluminum content (Al) |
| cellulose, sugar (D1490) [1] | **leaf calcium content (Ca)** |
| phenolic compounds, Tannin (D1660) [2] | **leaf iron content (Fe)** |
| phenolic compounds (D1460) [2] | **leaf potassium content (K)** |
| lignin (D1120) [1] | leaf magnesium content (Mg) |
| simple ratio index for the wavelength 680 nm (SR680) [3] | leaf manganese content (Mn) |
| simple ratio index for the wavelength 705 nm (SR705) [3] | leaf sulfur content (S) |
| modified chlorophyll absorption ratio index (mCARI) [4] | leaf C:N ratio |
| simple ratio index for the wavelength 705 nm (SR798) [3] |  |
| anthocyanin reflectance index (ARI) [5] |  |
| carotenoid related VI (ChappelleCar) [6] |  |
| carotenoid related VI (BlackburnCar1) [6] |  |
| carotenoid related VI (BlackburnCar2) [6] |  |
| carotenoid related VI (GitelsonCar1) [6] |  |
| carotenoid related VI (GitelsonCar2) [6] |  |
| leaf water vegetation index (LWVI 1) [7] |  |
| leaf water vegetation index (LWVI 2) [7] |  |
| water band index (WBI) [8] |  |
| water, starch (D970) [1] |  |
| water, cellulose, starch, lignin (D1200) [1] |  |
| water (D1400) [9] |  |
| **cadmium (D1240)** [10] |  |

All leaf traits of tree canopies, which were generated via a spectrometer or via conventional measurements in the field and the laboratory, that were gathered during the joint field campaign of the RESPECT project (Environmental Changes in Biodiversity Hotspot Ecosystems of South Ecuador: RESPonse and feedback effECTs). For detailed descriptions of variable generations see [11, 12]. Bold variables were selected for the analyses.

**References**

[1] Curran PJ. Remote sensing of foliar chemistry. *Remote Sens Environ* 1989; 30: 271–278.

[2] Kokaly RF, Skidmore AK. Plant phenolics and absorption features in vegetation reflectance spectra near 1.66 μm. *Int J Appl Earth Obs Geoinf* 2015; 43: 55–83.

[3] Sims DA, Gamon JA. Relationships between leaf pigment content and spectral reflectance across a wide range of species, leaf structures and developmental stages. *Remote Sens Environ* 2002; 81: 337–354.

[4] Mielke MS, Schaffer B, Schilling AC. Evaluation of reflectance spectroscopy indices for estimation of chlorophyll content in leaves of a tropical tree species. *Photosynthetica* 2012; 50: 343–352.

[5] Main R, Cho MA, Mathieu R, et al. An investigation into robust spectral indices for leaf chlorophyll estimation. *ISPRS J Photogramm Remote Sens* 2011; 66: 751–761.

[6] Meggio F, Zarco-Tejada PJ, Núñez LC, et al. Grape quality assessment in vineyards affected by iron deficiency chlorosis using narrow-band physiological remote sensing indices. *Remote Sens Environ* 2010; 114: 1968–1986.

[7] Galvão LS, Formaggio AR, Tisot DA. Discrimination of sugarcane varieties in Southeastern Brazil with EO-1 Hyperion data. *Remote Sens Environ* 2005; 94: 523–534.

[8] Ceccato P, Gobron N, Flasse S, et al. Designing a spectral index to estimate vegetation water content from remote sensing data: Part 1: Theoretical approach. *Remote Sens Environ* 2002; 82: 188–197.

[9] Penuelas J, Filella I, Biel C, et al. The reflectance at the 950-970 nm region as an indicator of plant water status. *Int J Remote Sens* 1993; 14: 1887–1905.

[10] Plaza S, Weber J, Pajonk S, et al. Wounding of Arabidopsis halleri leaves enhances cadmium accumulation that acts as a defense against herbivory. *BioMetals* 2015; 28: 521–528.

[11] Homeier J, Seeler T, Pierick K, et al. Leaf trait variation in species-rich tropical Andean forests. *Sci Rep* 2021; 11: 1–11.

[12] Limberger O, Homeier J, Farwig N, et al. Classification of Tree Functional Types in a Megadiverse Tropical Mountain Forest from Leaf Optical Metrics and Functional Traits for Two Related Ecosystem Functions. *Forests* 2021; 12: 649.
