# Supplementary material for: Effects of leaf traits of tropical trees on the abundance and body mass of herbivorous arthropod communities
Source: PLoS One. 2023 Nov 7;18(11):e0288276. doi: 10.1371/journal.pone.0288276 (PMC10629635; doi:10.1371/journal.pone.0288276)
Supplement: S4 Table — (DOCX) [file pone.0288276.s006.docx]

| **higher taxon** | **taxon** | **feeding guild** |
| --- | --- | --- |
| Arachnida | all | predators |
| Archaeognatha | all | saprobes |
| Blattodea | all | saprobes |
| Chilopoda | all | predators |
| Coleoptera | Adephaga | predators |
| Coleoptera | Chrysomeloidea | leaf chewer |
| Coleoptera | Coccinellidae, | predators |
| Coleoptera | Curculionoidea | rostrum chewers |
| Coleoptera | Staphylinidae | predators |
| Coleoptera | all remaining taxa | leaf chewer |
| Coleoptera | all larval stages | leaf chewer |
| Collembola | all | saprobes |
| Dermaptera | all | leaf chewer |
| Diplopoda | all | saprobes |
| Diptera | larval stages | predators |
| Diptera | adult stages | nectar suckers |
| Ephemeroptera | larval stages | unclear |
| Ephemeroptera | adult stages | non-feeding |
| Hemiptera | Auchenorrhyncha, | sap suckers |
| Hemiptera | Heteroptera (if piercing proboscis is not tight-fitting at rest and/or is convexly bent) | predators |
| Hemiptera | Heteroptera (all remaining) | sap suckers |
| Hemiptera | Sternorrhyncha, | sap suckers |
| Hymenoptera | Apocrita (adult stages) | predators |
| Hymenoptera | Symphyta (adult stages) | sap suckers |
| Hymenoptera | all larval stages | leaf chewers |
| Isopoda | all | saprobes |
| Lepidoptera | adult stages with working mouthparts | nectar suckers |
| Lepidoptera | adult stages without or with non-working mouthparts | non-feeding |
| Lepidoptera | all larval stages | leaf chewers |
| Mantodea | all | predators |
| Mecoptera | all larval stages | leaf chewers |
| Mecoptera | all adult stages | saprobes |
| Megaloptera | all larval stages | unclear |
| Megaloptera | all adult stages | non-feeding |
| Neuroptera | all | predators |
| Odonata | all | predators |
| Orthoptera | all | leaf chewer |
| Phasmatodea | all | leaf chewer |
| Phthiraptera | all | unclear |
| Psocoptera | all | saprobes |
| Raphidioptera | all | predators |
| Trichoptera | all | unclear |
| Thysanoptera | all | sap suckers |
| Zygentoma | all | saprobes |

**S4 Table.** Feeding guilds of larval and adult arthropod taxa.

Arthropod taxa used in the analyses were sorted according to the feeding guilds listed above. Feeding guilds were classified based on both literature and mouthparts.
