# Supplementary material for: Effects of leaf traits of tropical trees on the abundance and body mass of herbivorous arthropod communities
Source: PLoS One. 2023 Nov 7;18(11):e0288276. doi: 10.1371/journal.pone.0288276 (PMC10629635; doi:10.1371/journal.pone.0288276)
Supplement: S5 Table — (DOCX) [file pone.0288276.s007.docx]

| **model** | **fixed effects** | **estimate** | **(± SE)** | **p-value** |
| --- | --- | --- | --- | --- |
| **palatability ~ leaf traits** | | | | |
| log_10_(palatability + 0.00001) ~ log_10_(Ca) + log_10_(Fe) + log_10_(N) + SLA + D1420 + D1240 | intercept | -4.330 | (± 0.0940) | < 0.001 |
|  | log_10_(Ca) | -0.325 | (± 0.114) | 0.00564 |
|  | log_10_(Fe) | -0.212 | (± 0.101) | 0.0390 |
|  | log_10_(N) | 0.531 | (± 0.145) | < 0.001 |
|  | SLA | -0.378 | (± 0.139) | 0.00786 |
|  | D1420 | 0.285 | (± 0.128) | 0.0285 |
|  | D1240 | 0.288 | (± 0.122) | 0.0208 |
| **palatability ~ leaf traits + elevation** | | | | |
| log_10_(palatability + 0.00001) ~ log_10_(Ca) + log_10_(K) + log_10_(Fe) + log_10_(N) + site + D1240 | intercept | -4.76 | (± 0.181) | < 0.001 |
|  | log_10_(Ca) | -0.274 | (± 0.122) | 0.0272 |
|  | log_10_(K) | -0.200 | (± 0.0926) | 0.0336 |
|  | log_10_(Fe) | -0.204 | (± 0.102) | 0.0487 |
|  | log_10_(N) | 0.355 | (± 0.114) | 0.00255 |
|  | site | 0.726 | (± 0.274) | 0.00977 |
|  | D1240 | 0.238 | (± 0.116) | 0.0439 |

**S5 Table. Minimal adequate linear models showing the relations between leaf traits and palatability.**

Included to the models were the 16 pre-selected leaf traits measured during the joint field campaign from February to March 2019. For explanations of variable abbreviations see S3 Table. Palatability was estimated experimentally in a feeding trial with the cricket *Gryllus assimilis* (Orthoptera) and is determined as consumed dry mass [g] per tree. SE = standard error, log_10_(x) = base 10 logarithmized. Significance was defined at a 5% level.
