# Supplementary material for: Effects of leaf traits of tropical trees on the abundance and body mass of herbivorous arthropod communities
Source: PLoS One. 2023 Nov 7;18(11):e0288276. doi: 10.1371/journal.pone.0288276 (PMC10629635; doi:10.1371/journal.pone.0288276)
Supplement: S6 Table — (DOCX) [file pone.0288276.s008.docx]

**S6 Table.** GLMM models showing the effects of leaf traits on chewer abundance per elevation.

| **model** | **fixed effects** | **estimate** | **SE** | **p-value** |
| --- | --- | --- | --- | --- |
| **leaf chewers** | | | | |
| **at 1000 m a.s.l.** | | | | |
| abundance ~ D1420 + log_10_(Ca) + log_10_(K) + D1240 + log_10_(N) + log_10_(DBH) + log_10_(Fe) +  log_10_(P) + SLA + (1 \| family:genus) | intercept | 3.71 | 0.247 | < 0.001 |
|  | D1420 | 0.0938 | 0.0421 | 0.026 |
|  | log_10_(Ca) | 0.524 | 0.0467 | < 0.001 |
|  | log_10_(K) | -0.0561 | 0.0215 | 0.00916 |
|  | D1240 | 0.462 | 0.0686 | < 0.001 |
|  | log_10_(N) | 1.19 | 0.0672 | < 0.001 |
|  | log_10_(DBH) | 0.19 | 0.0146 | < 0.001 |
|  | log_10_(Fe) | -0.0479 | 0.0228 | 0.0351 |
|  | log_10_(P) | -0.288 | 0.0317 | < 0.001 |
|  | SLA | 0.265 | 0.0458 | < 0.001 |
|  | **random effects** | **variance** | **SD** |  |
|  | family:genus | 0.898 | 0.947 |  |
| **at 2000 m a.s.l.** | | | | |
| abundance ~ D1420 + D1240 + log_10_(P) + log_10_(N) + log_10_(DBH) + SLA + log_10_(Ca) + log_10_(K) + (1 \| plot) + (1 \| family:genus) | intercept | 5.56 | 0.252 | < 0.001 |
|  | D1420 | -0.171 | 0.0264 | < 0.001 |
|  | D1240 | -0.139 | 0.0218 | < 0.001 |
|  | log_10_(P) | -0.441 | 0.051 | < 0.001 |
|  | log_10_(N) | 0.203 | 0.0569 | < 0.001 |
|  | log_10_(DBH) | 0.113 | 0.0196 | < 0.001 |
|  | SLA | -0.141 | 0.0311 | < 0.001 |
|  | log_10_(Ca) | -0.147 | 0.0319 | < 0.001 |
|  | log_10_(K) | 0.333 | 0.0749 | < 0.001 |
|  | **random effects** | **variance** | **SD** |  |
|  | family:genus | 0.141 | 0.376 |  |
|  | plot | 0.159 | 0.399 |  |
| **rostrum chewers** | | | | |
| **at 1000 m a.sl.** | | | | |
| abundance ~ log_10_(K) + log_10_(P) + log_10_(DBH) + SLA + (1 \| plot) + (1 \| family:genus) | intercept | 3.25 | 0.291 | < 0.001 |
|  | log_10_(K) | 0.178 | 0.0561 | 0.00139 |
|  | log_10_(P) | -0.284 | 0.0885 | 0.00133 |
|  | log_10_(DBH) | -0.143 | 0.0365 | < 0.001 |
|  | SLA | 0.204 | 0.112 | 0.0699 |
|  | **random effects** | **variance** | **SD** |  |
|  | family:genus | 0.176 | 0.419 |  |
|  | plot | 0.199 | 0.446 |  |
| **at 2000 m a.s.l.** | | | | |
| abundance ~ D1240 + log_10_(DBH) + SLA + log_10_(N) + (1 \| family:genus) | intercept | 3.56 | 0.135 | < 0.001 |
|  | D1240 | -0.162 | 0.0333 | < 0.001 |
|  | log_10_(DBH) | 0.102 | 0.0356 | 0.00406 |
|  | SLA | 0.18 | 0.0569 | 0.00159 |
|  | log_10_(N) | -0.168 | 0.07 | 0.0162 |
|  | **random effects** | **variance** | **SD** |  |
|  | family:genus | 0.246 | 0.496 |  |

Minimal adequate model results of the GLMMs of chewer abundance per feeding guild and leaf traits at 1000 m a.s.l. and at 2000 m a.s.l. Model construction and reduction was the same as for the LME including both elevation levels described in the main text. SE = standard error, SD = standard deviation, log_10_(x) = base 10 logarithmized. Significance was defined at a 5% level.
