# Supplementary material for: Effects of leaf traits of tropical trees on the abundance and body mass of herbivorous arthropod communities
Source: PLoS One. 2023 Nov 7;18(11):e0288276. doi: 10.1371/journal.pone.0288276 (PMC10629635; doi:10.1371/journal.pone.0288276)
Supplement: S7 Table — (DOCX) [file pone.0288276.s009.docx]

**S7 Table.** LME models showing the effects of leaf traits on mean body mass [g] of chewers per elevation.

| **model** | **variable** | **estimate** | **SE** | **p-value** |
| --- | --- | --- | --- | --- |
| **leaf chewers** | | | | |
| **at 1000 m a.s.l.** | | | | |
| log_10_(mBM) ~ (1 \| plot) + (1 \| family:genus:species) | none |  |  |  |
|  | **random effects** | **variance** | **SD** |  |
|  | genus:family | 5.29e^-03^ | 7.27e^-02^ |  |
|  | species:(genus:family) | 8.28e^-04^ | 2.88e^-02^ |  |
|  | family | 4.28e^-10^ | 2.07e^-05^ |  |
|  | plot | 0.0 | 0.0 |  |
| **at 2000 m a.s.l.** | | | | |
| log_10_(mBM) ~ (1 \| plot) + (1 \| family:genus:species) | none |  |  |  |
|  | **random effects** | **variance** | **SD** |  |
|  | genus:family | 0.0 | 0.0 |  |
|  | species:(genus:family) | 0.0 | 0.0 |  |
|  | family | 0.0 | 0.0 |  |
|  | plot | 0.0156 | 0.125 |  |
| **rostrum chewers** | | | | |
| **at 1000 m a.s.l.** | | | | |
| log_10_(mBM) ~ log_10_(K) + (1 \| plot) + (1 \| family:genus:species) | intercept | -2.64 | 0.0437 | < 0.001 |
|  | log_10_(K) | -0.103 | 0.032 | 0.00249 |
|  | **random effects** | **variance** | **SD** |  |
|  | genus:family | 0.0 | 0.0 |  |
|  | species:(genus:family) | 0.0 | 0.0 |  |
|  | family | 0.0 | 0.0 |  |
|  | plot | 0.0 | 0.0 |  |
| **at 2000 m a.s.l.** | | | | |
| log_10_(mBM) ~ log_10_(P) + SLA + (1 \| plot) + (1 \| family:genus:species) | intercept | -2.75 | 0.0996 | < 0.001 |
|  | log_10_(P) | 0.154 | 0.061 | 0.0161 |
|  | SLA | -0.15 | 0.0705 | 0.0401 |
|  | **random effects** | **variance** | **SD** |  |
|  | genus:family | 1.82e^-10^ | 1.35e^-05^ |  |
|  | species:(genus:family) | 0.0 | 0.0 |  |
|  | family | 5.976e^-03^ | 0.0773 |  |
|  | plot | 1.72e^-02^ | 0.131 |  |

Minimal adequate model results of the LMEs of mean body mass per feeding guild and leaf traits. Model construction and reduction was the same as for the LME including both elevation levels described in the main text. mBM = mean body mass, SE = standard error, SD = standard deviation, log_10_(x) = base 10 logarithmized. Significance was defined at a 5% level.
