# Supplementary material for: Effects of leaf traits of tropical trees on the abundance and body mass of herbivorous arthropod communities
Source: PLoS One. 2023 Nov 7;18(11):e0288276. doi: 10.1371/journal.pone.0288276 (PMC10629635; doi:10.1371/journal.pone.0288276)
Supplement: S1 File — (DOCX) [file pone.0288276.s012.docx]

**S1 File. Variable selection.**

To select a reliable subset of variables that were collected during our joined field campaign (S3 Table, S1 Figure A), we excluded all spectrally measured traits based on wavelengths that could represent more than one chemical (D1690, D1490, D970, and D1200). Further, we excluded all spectrally estimated traits that represented the water content of a leaf (WBI, D1400, SR789, and LWVI 1 and 2), since it can be highly variable in the field. That makes it difficult to draw relations to herbivore abundance and mean body mass. Additionally, spectrally measured traits related to chlorophyll content (SR680, SR705, and mCARI), the greenness of a leaf (vis and nir albedo, NDVI, EVI, RGVI, and RBVI), and carotenoids (ChappelleCar, BlackburnCar 1 and 2, and GitelsonCar 1 and 2), which are known to protect chlorophyll from UV radiation [1], were removed, as these traits seem to have no direct impact on palatability. Further, the unspecific spectral traits correlated with oil (D1040) and phenolic compounds (D1460) were eliminated, since interpretation would be difficult. Regarding the conventionally estimated leaf traits, we excluded sulfur for which we found little evidence in literature to be related to herbivorous arthropods. Leaf thickness and toughness as well as leaf area were intercorrelated with SLA (S1 Figure A) and since SLA is an often-used variable in the context of herbivorous arthropods [e.g. 32, 37, 45, 47, 52], we excluded the other three variables from the analyses. Regarding the variables N, P, N:P ratio, C:N ratio, and the spectrally measured NDNI, we kept the N- and P concentrations in the analyses and excluded NDNI and the two ratios, since these five variables were intercorrelated with each other and interpretations of directly measured nutrient concentrations are more straightforward than of ratios and indices. For the remaining leaf traits (S1 Figure 2), we found evidence of impacts on herbivorous arthropods in literature (see S1 Table and [7–12]). However, to build valid models, we did not want to exceed the number of nine leaf traits used in the models, since we have 90 datapoints. To try to further reduce the number of variables, we examined, if these variables can be grouped into principal components by applying a phylogenetic principal component analysis (pPCA). Therefore, we built a phylogenetic tree for the 30 tree species used in our analyses based on the backbone megatree S03_R20160415_euphyllophyte.new and the corresponding ages calibration data S12_ages_magallon_PL_euphyllophyte, which were personally provided by M. Gastauer and J. Meira-Neto. The provided data are based on [13]. We built our uncalibrated tree, with tree species at the branch tips, with the online tool Phylomatic version 3 (https://phylodiversity.net/phylomatic/ [14]) and calibrated it afterwards in R version 4.0.2 [15] by using the packages *phytools* [16] and *phylocomr* [17]. To run a pPCA with the remaining traits: SLA, DMC, C, N, P, Al, Ca, Fe, K, Mg, Mn, DBH, D1420, D1660, D1240, and ARI with the package *phytools* [16] (S2 Figure A), we square root transformed Mg and base 10 logarithmized the variables N, P, Al, Ca, Fe, K, Mn, and DBH to control for normal distribution. Additionally, all variables were z-transformed due to very different scales. Identifying the number of significant principal components (PCs) by applying the broken stick method with the package *PCDimension* [18] resulted in no significant PC (S2 Figure B). Additionally, no variable grouping within and between PCs was identifiable. Therefore, we used single variables for our analyses and had to further reduce the number of variables to build reliable models.

Thus, as a next step, we analyzed which leaf traits of our pre-selected traits were significantly correlated with the experimentally estimated palatability to test, if palatability is an appropriate measure to be related to chewer abundance and mean body mass and to identify those leaf traits that can be used in the following analyses. Therefore, we applied two linear mixed-effects models. One model was built to examine relations between leaf traits and palatability independently from defining differences within traits as related to elevation. Therefore, we used (1) the experimentally estimated base 10 logarithmized palatability as response variable and the z-transformed remaining base 10 logarithmized, or square root-transformed leaf traits as predictor variables. Additionally, the 100 m x 100 m plots (‘plot’), where the trees were sampled were added as random effect to account for pseudoreplication. To consider the tree phenology, species nested within genus, nested within family was included as random effect. Additionally, we built a second linear mixed-effects model, which considered the elevation as driver for variation within leaf traits to analyze if the significance of relationships between leaf traits and palatability varied depending on the elevation level the tree grew. Therefore, we (2) built the same model as for (1) and added the variable (‘site’) as fixed effect which determined at which elevation the tree occurred.

To define the minimal adequate models, we excluded non-significant predictor variables, by applying automated stepwise backward selections, based on models, which converged with as many independent variables as possible. Both minimal adequate and maximum models were calculated using the R package *buildmer* [19], considering the numerical optimization algorithm ‘bound optimization by quadratic approximation’. After the reduction of the first model – without the consideration of the elevation – the leaf traits Ca, Fe, N, SLA, D1420 (lignin), and D1240 (cadmium) were significantly related to palatability (S4 Table). When including the elevation as fixed effect, the minimal adequate model revealed that Ca, Fe, N, D1240 (cadmium), K, and elevation at 2000 m were significantly related to palatability (S4 Table).

Referring to these results, we chose the following leaf traits for our analyses: Fe, D1240 (cadmium), Ca, N, SLA, K, D1420 (lignin), and elevation. Since P forms an important part of several cellulose biochemicals, like RNA, which in turn is positively related to growth rates [20], we included this trait in our analyses, although it was not significantly related to palatability. Additionally, we included the plant trait DBH as covariate in our analyses, since it is positively related to above-ground tree biomass production and thus to canopy size [21, 22], which could affect arthropod abundance and mean body mass.

**S1 Figure.** **Correlation matrices of leaf traits.**

A) Correlation matrix of 48 leaf traits measured spectrally or with conventional measurement techniques in the field and the laboratory and which were collected from trees during a joined field campaign from February to March 2019. For detailed descriptions of the abbreviations of the leaf traits see S3 Table. B) Correlation matrix of the 16 pre-selected leaf traits after a first variable reduction. Correlation intensity ranges from red = highly negatively correlated over white = not correlated to blue = highly positively correlated.

**S2 Figure.** **Phylogenetic principal component analysis of the similarity of various leaf traits.**

A) shows the ordination diagram of the phylogenetic principal component analysis (pPCA) dealing with the (dis)similarity of the 16 preselected leaf traits (red vectors) from S1 Figure B. Leaf traits were base 10- or square root transformed, if corrections for a normal distribution were necessary. All leaf traits were z-transformed. For detailed descriptions of the abbreviations of the leaf traits see S3 Table. The 90 tree individuals used in the analyses are represented as black dots. PC = principal component, lg = base 10 logarithmized, sqrt = square root transformed. B) shows the eigenvalues for each PC of the pPCA. The PC1 explained 16.4 % of the variance, the PC2 explained 15.2 %, PC3 = 13.8 %, PC4 = 10.5 %, PC5 = 8.3 %, PC6 = 6.8 %, PC7 = 5.5 %, PC8 = 4.7 %, PC9 = 4.1 %, PC10 = 3.9 %, PC11 = 2.7 %, PC12 = 2.6 %, PC13 = 2.0 %, PC14 = 1.8 %, PC15 = 1.0 %, and PC16 = 0.8 % of the variance. No PC was significant.

**References**

[1] Heath JJ, Cipollini DF, Stireman JO. The role of carotenoids and their derivatives in mediating interactions between insects and their environment. *Arthropod Plant Interact* 2013; 7: 1–20.

[2] Descombes P, Marchon J, Pradervand JN, et al. Community-level plant palatability increases with elevation as insect herbivore abundance declines. *J Ecol* 2017; 105: 142–151.

[3] Coley PD. Herbivory and Defensive Characteristics of Tree Species in a Lowland Tropical Forest. *Ecol Soc Am* 1983; 53: 209–233.

[4] Pérez-Harguindeguy N, Díaz S, Vendramini F, et al. Leaf traits and herbivore selection in the field and in cafeteria experiments. *Austral Ecol* 2003; 28: 642–650.

[5] Poorter L, Van De Plassche M, Willems S, et al. Leaf traits and herbivory rates of tropical tree species differing in successional status. *Plant Biol* 2004; 6: 746–754.

[6] Wang MQ, Li Y, Chesters D, et al. Host functional and phylogenetic composition rather than host diversity structure plant–herbivore networks. *Mol Ecol* 2020; 29: 2747–2762.

[7] Pilon-Smits EA, Quinn CF, Tapken W, et al. Physiological functions of beneficial elements. *Curr Opin Plant Biol* 2009; 12: 267–274.

[8] Ribeiro SP, Londe V, Bueno AP, et al. Plant defense against leaf herbivory based on metal accumulation: examples from a tropical high altitude ecosystem. *Plant Species Biol* 2017; 32: 147–155.

[9] Awmack CS, Leather SR. Host Plant Quality and Fecundity in Herbivorous Insects. *Annu Rev Entomol* 2002; 47: 817–844.

[10] Martinek P, Hedbávný J, Kudláček T, et al. Adverse responses of Cabera pusaria caterpillars to high dietary manganese concentration. *Entomol Exp Appl* 2020; 168: 635–643.

[11] Gong WC, Liu YH, Wang CM, et al. Why Are There so Many Plant Species That Transiently Flush Young Leaves Red in the Tropics? *Front Plant Sci* 2020; 11: 1–12.

[12] Malakar C, Ganguly A, Haldar P. Influence of cadmium on growth, survival and clutch size of a common Indian short horned grasshopper, Oxya fuscovittata. *Am J Toxicol Sci* 2009; 1: 32–36.

[13] Gastauer M, Meira-Neto JAA. Updated angiosperm family tree for analyzing phylogenetic diversity and community structure. *Acta Bot Brasilica* 2017; 31: 191–198.

[14] Webb CO, Donoghue. MJ. Phylomatic: tree assembly for applied phylogenetics. *Mol Ecol Notes* 2005; 5: 181–183.

[15] R Core Team. *R: A language and environemnt for statistical computing. R Foundation for Statistical Computing*. Vienna, Austria, https://www.r-project.org/. (2020).

[16] Revell LJ. phytools: An R package for phylogenetic comparative biology (and other things). *Methods Ecol Evol* 2012; 3: 217–223.

[17] Ooms J, Chamberlain S. phylocomr: Interface to ‘Phylocom’. R package version 0.3.2., https://cran.r-project.org/package=phylocomr (2019).

[18] Coombes KR, Wang M. PCDimension: Finding the Number of Significant Principal Components. R package version 1.1.11., https://cran.r-project.org/package=PCDimension%0A (2019).

[19] Voeten CC. buildmer: Stepwise Elimination and Term Reordering for Mixed-Effects Regression. R package version 1.9., https://cran.r-project.org/package=buildmer (2021).

[20] Elser JJ, Dobberfuhl DR, MacKay NA, et al. Organism size, life history and N:P stoichiometry. *Bioscience* 1996; 46: 674–685.

[21] Haase R, Haase P. Above-ground biomass estimates for invasive trees and shrubs in the Pantanal of Mato Grosso, Brazil. *For Ecol Manage* 1995; 73: 29–35.

[22] Chave J, Andalo C, Brown S, et al. Tree allometry and improved estimation of carbon stocks and balance in tropical forests. *Oecologia* 2005; 145: 87–99.
